# *CREBBP* knockdown suppressed proliferation and promoted chemo-sensitivity via PERK-mediated unfolded protein response in ovarian cancer

**DOI:** 10.7150/jca.56135

**Published:** 2021-06-01

**Authors:** Haoyang Hu, Sheng Yin, Ruyue Ma, Rujun Chen, Shuqing Li, Yaping Chen, He Fei, Lina Yang

**Affiliations:** 1 Department of Obstetrics and Gynecology, Shanghai Fifth People's Hospital, Fudan University, 801 Heqing Road, Shanghai, People's Republic of China.; 2 Department of Obstetrics and Gynecology, Zhongshan Hospital Fudan University, 180 Fenglin Road, Shanghai, People's Republic of China.; 3 Obstetrics and Gynecology Hospital of Fudan University, 128 Shenyang Road, Shanghai, People's Republic of China.

**Keywords:** CREBBP, UPR, chemo-sensitivity, apoptosis, ovarian cancer

## Abstract

CREBBP, in short CBP, has been reported to be involved in tumorigenesis in various cancers, but its role in ovarian cancer remains largely unexplored. In our study, survival analysis of CBP in patients with ovarian cancer was conducted using the Kaplan-Meier Plotter database, then we utilized specific shRNA targeting *CREBBP* to block the expression of CBP, and detected its effect on cell proliferation and chemo-sensitivity in ovarian cancer cells. The results showed that high expression of CBP was correlated with poor prognosis in ovarian cancer patients. *CREBBP* knockdown in ovarian cancer cells significantly inhibited tumor proliferation both *in vitro* and *in vivo*. Moreover, *CREBBP* knockdown promoted chemo-sensitivity in ovarian cancer cells. Mechanism research further demonstrated that *CREBBP* knockdown attenuated unfolded protein response (UPR), which was mediated by PERK/ATF4/STC2 signaling pathway. Our research linked CBP and UPR in ovarian cancer and may provide new strategies for the clinical treatment of ovarian cancer.

## Introduction

Ovarian cancer is one of the five leading causes of cancer deaths among American women 1 , and also the 8^th^ leading cause of death worldwide in 2020 2. Epithelial ovarian cancer (EOC) accounts for over 95% of ovarian malignancies, and high grade serous ovarian cancer is the most common histologic subtype, accounting for over 70% of EOC 3. EOC generally presents at an advanced stage with the 5-year overall survival rate from 30% to 40% 4. It is urgent to develop new strategies for early diagnosis and treatment of ovarian cancer.

The CREB-binding protein (CREBBP, in short CBP) is a transcriptional co-activator with lysine acetyltransferase activity. CBP has been reported to be involved in various cellular processes including cell metabolism, embryonic development and cell differentiation 5-8. Previous research on the role of CBP in tumorigenesis were mainly derived from gene mutation or chromosome arrangement 9-11. Recently, studies to investigate the function of CBP on epigenetic modification or directly acetylation have attracted more and more attention. CBP and PTEN knockdown alone or together could increase the H3K27me3 levels and inhibit H3K27Ac in prostate cancer 12. *Crebbp* knockdown in small cell lung cancer cells reduced H3K27Ac levels leading to epigenetic suppression of *CDH1* and other cell adhesion-associated genes 13. CBP directly acetylated BCAT2 to promote protein degradation 14, CBP dramatically enhanced DOT1L acetylation and promoted protein stability 15 and CBP directly acetylated KDM2B and promoted target gene transcription 16. The aforementioned study demonstrated that CBP could serve as a tumor suppressor or an oncogene in different cancer types. Till now, the role of CBP in ovarian cancer, especially in epithelial ovarian cancer has not yet been fully elucidated.

Endoplasmic reticulum (ER) is an organelle for protein folding. Once misfolded proteins or unfolded proteins accumulated, the unfolded protein response (UPR) is triggered to maintain ER homeostasis and protect cell from death. Generally, The UPR is mediated by three ER transmembrane sensors: PERK, IRE1α and ATF6α 17. PERK activates eIF2α phosphorylation to attenuate global protein translation but selectively enhances ATF4 and subsequently downstream effectors such as STC2 18; IRE1α mediates cleavage of X-box binding protein (*uXBP1*) mRNA into spliced *XBP1* (*sXBP*1) mRNA, which is translated into a potent transcriptional activator that induces the expression of UPR responsive genes; ATF6α dissociates from GRP78 and translocates to the Golgi where ATF6α is cleaved and activated, the activated ATF6α translocates to the nucleus and activates the transcription of target genes. The three signaling pathways are intertwined to promote cell survival 19-21.

In the present study, we utilized shRNA targeting *CREBBP* to inhibit CBP expression and examined its effect on ovarian cancer cells. The results showed that *CREBBP* knockdown suppressed tumor proliferation and promoted chemo-sensitivity via mitigating the PERK-mediated unfolded protein response (UPR). Our results may provide new cues for the clinical treatment of ovarian cancer.

## Materials and methods

### Reagents and antibodies

RPMI-1640 cell culture medium and DMEM cell culture medium were purchased from Sigma-Aldrich Inc. (St. Louis, MO, USA). Fetal bovine serum (FBS) and Plasmocin™ were purchased from Thermo Fisher Scientific (Waltham, MA, USA). LipoFiter^TM^ Liposomal Transfection Reagent was purchased from Hanbio Biotechnology (Shanghai, China). CDDP was purchased from Selleck Chemicals (Houston, TX, USA). Antibodies used were listed in Table 1. HRP-conjugated goat anti-rabbit and anti-mouse secondary antibodies were from Sigma-Aldrich Inc. (St. Louis, MO, USA). EnVision^TM^ Ⅲ Detection System (GK500705) was purchased form Gene Tech (Shanghai, China).

### Cell lines and cell culture

Human ovarian cancer cell lines (HEY and SKOV3) and human embryonic kidney 293T cell line were obtained from ATCC. Ovarian cancer cells were cultured in RPMI-1640 medium supplemented with 10% FBS. 293T cells were grown in DMEM medium with 10% FBS. All the cells were cultured at 37 °C in a 5% CO_2_ humidified incubator. STR DNA profiling was used for cell line authentication. Plasmocin™ was used for removal of mycoplasma in cell culture.

### Construction of *CREBBP* knockdown plasmid

The pLKO.1 - TRC cloning vector was obtained from Addgene (Cambridge, MA, USA). shRNA target sequences are as follows: shCBP-1: GCGGAGCCATCTAGTGCATA; ShCBP-2: GCAAGACATCCCGAGTCTATA; shLuc (Ctrl): CTTACGCTGAGTACTTCGA. The experiment was conducted according to the Addgene's protocol. The positive clones were verified by sequencing.

### Establishment of *CREBBP* knockdown cells

The pLKO.1 construct was co-transfected into 293T cells with psPAX2 and pMD2.G by LipoFiter^TM^ (Hanbio, Shanghai, China). The supernatants containing virus particles were harvested after transfection for 48h, and filtered through a 0.45 μm filter to remove the cell debris. Then the virus particles were used to infect cancer cells along with polybrene (8 μg/mL). Subsequently, stable cell lines were selected with 1 μg/mL puromycin for 2 weeks.

### qRT-PCR

Total RNA was extracted using TRIzol reagent (Thermo Fisher Scientific, Waltham, MA, USA). Then 1 μg of total RNA was reverse transcribed into cDNA using the PrimeScript RT Reagent Kit with gDNA Eraser (Takara, Dalian, China). qRT-PCR was performed using the FastStart Universal SYBR Green Master (Roche Diagnostics, Indianapolis, IN, USA) in the Applied Biosystems 7500 Real-Time PCR System. The primer sets used were listed as follows: sense primer for *CREBBP,* 5′-CGGCTCTAGTATCAACCCAGG-3′, and antisense primer for *CREBBP*, 5′-TTTTGTGCTTGCGGATTCAGT-3′; sense primer for *GAPDH*, 5′-TTGAGGTCAATGAAGGGGTC-3′ and antisense primer for *GAPDH*, 5′-GAAGGTGAAGGTCGGAGT A-3′. Relative mRNA levels were analyzed using the Comparative Ct Threshold Method (ΔΔCT) with *GAPDH* as a reference gene.

### CCK-8 assay for cell proliferation

The indicated cells were seeded at a density of 700 cells per well in 96-well plates. Then the cells were incubated with 10 μL CCK-8 per well for 2 h at the same time on day 1, 2, 3, 4 and 5. The plates were then shaken for 10 min, and the optical density (OD) at 450 nm was measured using a microplate reader. Each sample was analyzed three times in quintuplicate.

### Colony formation assay

Cells were plated in 6-well plates at a density of 500 cells per well and allowed to grow for 10 days to form obvious clones. Then the cells were stained with 0.1% crystal violet in methanol. After washed with double distilled water, the clones were imaged, and the numbers of clones were quantified and analyzed using Graphpad Prism version 8.0.2 (GraphPad Software Inc., San Diego, CA, USA). Each experiment was repeated three times.

### IC50 measurement

The indicated cells were seeded at a density of 6000 cells per well in 96-well plates and incubated overnight. CDDP was added at a concentration of 0, 1, 2, 4, 8, 16 and 32 μM respectively. After 48 h, the cells were incubated with fresh medium containing 10 μL CCK-8 per well for 2 h. The plates were then shaken for 10 min, and the optical density (OD) at 450 nm was measured using a microplate reader. Each sample was analyzed three times in quadruplicate.

### Examination of apoptosis

Cells were plated in 6 cm dishes and incubated overnight. Then CDDP was added into the medium. After incubation for 48h, cells were collected by trypsinization and stained by the Annexin V-FITC/PI Apoptosis Detection Kit (YEASEN Biotechnology, Shanghai, China) according to the manufacturer's instruction. The samples were examined by using the Amnis FlowSight^®^ Imaging Flow Cytometer, and analyzed by the IDEAS software. The experiment was repeated three times.

### Western blot assay

Whole cell lysates were generated using RIPA lysis buffer (Beyotime Biotechnology, Jiangsu, China) and quantified by the BCA Protein Assay Kit (Beyotime Biotechnology, Jiangsu, China). Protein samples were separated in SDS-PAGE and transferred onto PVDF membrane. Then the membrane was blocked with 5% non-fat milk for 1h at room temperature, and incubated with the indicated primary antibodies at 4 °C overnight with gently shaking. After washing in TBST, the membrane was incubated with HPR-conjugated secondary antibody for 1h at room temperature. The protein bands were visualized using the enhanced chemiluminescence substrate kit (Millipore, Schwalbach, Germany) in the FluorChem E system.

### *XBP1* Splicing Assay

RNA extraction and cDNA synthesis were performed as described in qRT-PCR. *XBP1* cDNA was amplified by PCR with the PrimeSTAR GXL DNA Polymerase (Takara, Dalian, China). The primer set encompassing the spliced sequences of human* XBP1* was as follows: sense primer, 5′-AAACAGAGTAGCAGCTCAGACTGC-3′; antisense primer, 5′-TCCTTCTGGGTAGACCTCTGGGAG-3′. The amplification products were further digested by *Pst*I at 37 °C for 1h, and subsequently separated by DNA electrophoresis on a 2% agarose gel containing Gelred (Biotium Inc, Heyward, California, USA). The primer set for *GAPDH* amplification was described in qRT-PCR.

### Xenograft model in nude mice

The xenograft models were generated in female athymic nude mice. The assay was carried out with the institutional guidelines and approved by ECNU Multifunctional Platform for Innovation (011). Cells were harvested by trypsinization and resuspended in 1×PBS, then subcutaneously injected into 6-week-old BALB/c athymic nude mice. Six mice were used per cell lines, each mouse received twice injection in bilateral flank and each injection contained 5×10^6^ cells. Tumor growth was monitored until the end point, and tumor size was measured every 3 days using an electronic caliper. At the termination of the study (the 27^th^ day), the mice were euthanized with CO_2_ exposure and necropsied to collect the subcutaneous tumors. Tumor volume was calculated with the formula: V=L×W×H×0.52.

### Immunohistochemistry

The subcutaneous xenograft tumors were dissected and fixed in formalin. After dehydration and paraffin-embedded, the tumors were sliced into 5 μm thickness sections, and the sections were deparaffinized with xylene, rehydrated in graded alcohol solutions. One set of slides was stained with hematoxylin and eosin. Other two sets of slides were stained with antibodies against CBP and PCDA respectively according to the manufacturer's instruction. Briefly, endogenous peroxidase activity was quenched using 3% H_2_O_2_ in methanol for 15 minutes at room temperature, antigen retrieval was performed by boiling the slides in 0.01M citrate buffer (pH 6.0). After blocking in 3% BSA for 1h at room temperature, the slides were incubated with antibodies against CBP (1:100) and PCNA (1:1000) respectively at 4 °C overnight and incubated with secondary antibodies conjugated HRP for 1h at room temperature. All slides were developed with DAB chromogen supplied by the EnVision^TM^ Ⅲ Detection System and counterstained with hematoxylin. After dehydration in graded alcohol solutions and transparence in xylene, the slides were mounted with neutral gum and analyzed under a brightfield microscope.

### Statistical analysis

Data were presented as mean ± standard deviation (SD). Data between two groups were analyzed by student's *t* test (two-tailed distribution). Data between three groups were analyzed by one-way ANOVA, followed by Dunnett's multiple comparisons test. Statistical significance is described as follows: * P < 0.05, ** P <0.01 and *** P <0.001. P < 0.05 was considered statistically significant. Statistical analysis and graph production were performed using Graphpad Prism version 8.0.2 (GraphPad Software Inc., San Diego, CA, USA).

## Results

### High expression of CBP is correlated with poor prognosis in ovarian cancer patients

**Figure 1:**
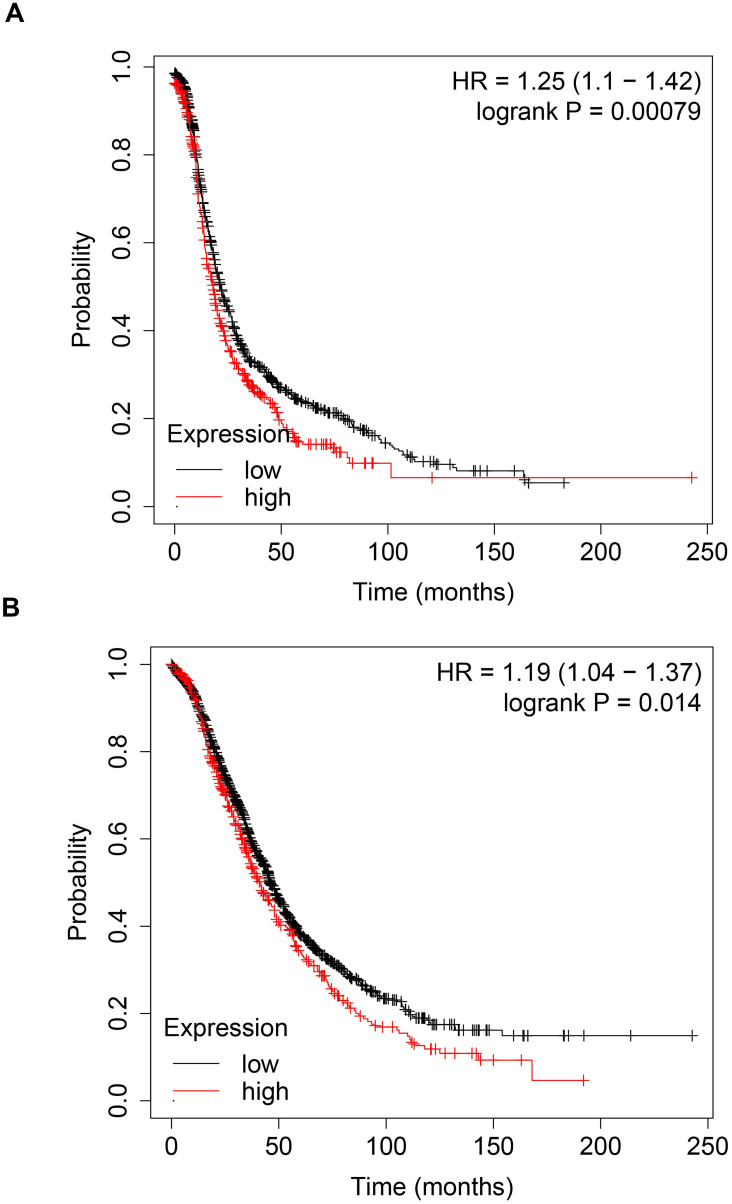
** High expression of CBP predicts unfavorable prognosis in patients with ovarian cancer. A.** High expression of CBP predicts poor PFS in patients with ovarian cancer. **B.** High expression of CBP predicts unfavorable OS in patients with ovarian cancer. PFS, progression free survival; OS, overall survival; HR, hazard ratio.

To explore the prognostic value of CBP in patients with ovarian cancer, we analyzed the prognosis data in the Kaplan-Meier plotter database (http://kmplot.com/analysis/). As shown in Fig. 1A, high expression of CBP was associated with poor PFS (Fig. 1A, p = 0.00079, HR = 1.25). Furthermore, high expression of CBP also predicted poor OS in ovarian cancer patients (Fig. 1B, p = 0.014, HR = 1.19). Taken together, these data suggested that CBP could serve as a promising prognostic marker in ovarian cancer.

### *CREBBP* knockdown inhibits cell proliferation in ovarian cancer cells

**Figure 2:**
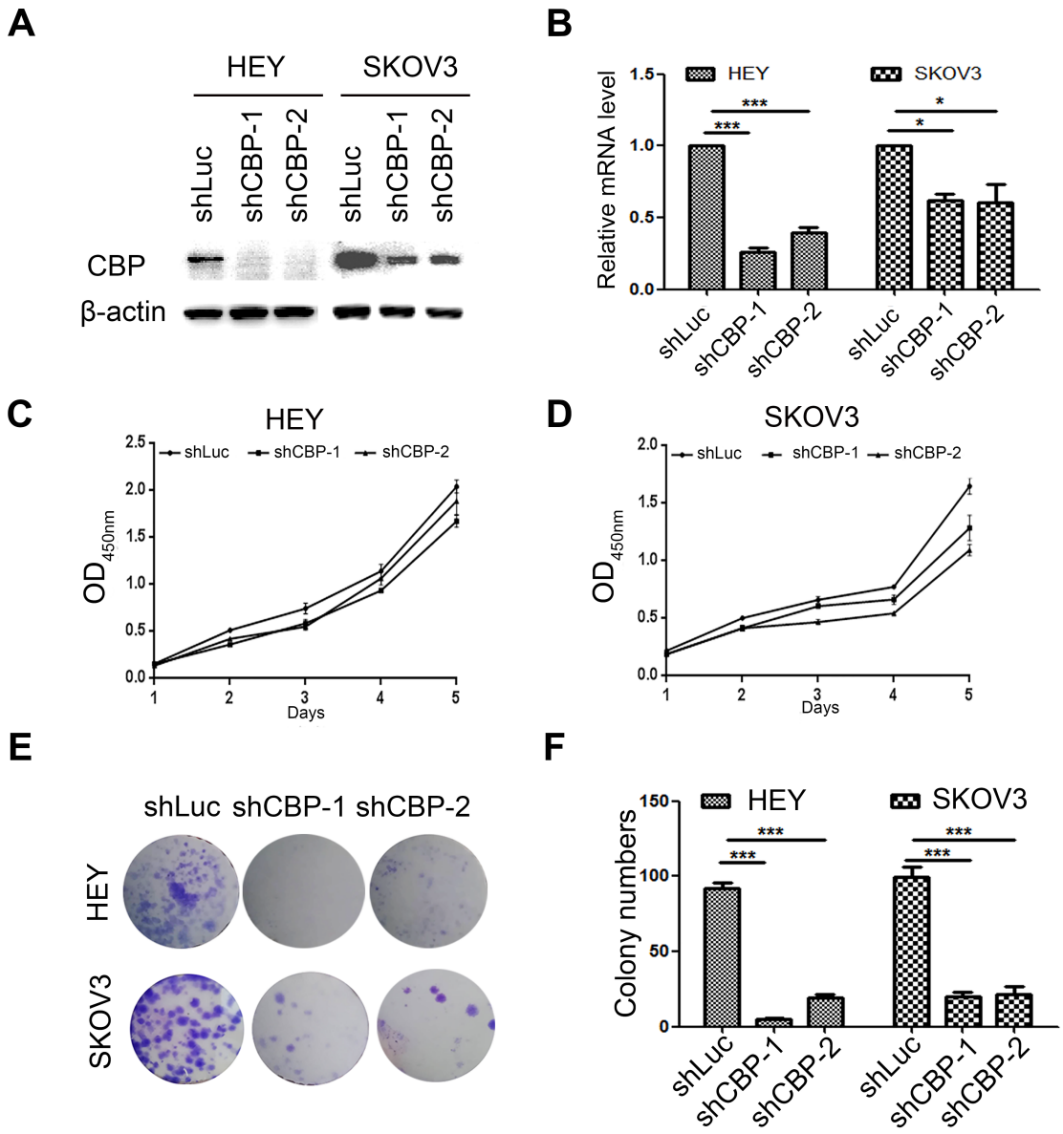
***CREBBP* knockdown inhibits proliferation in ovarian cancer cells. A.** Validation of *CREBBP* knockdown by western blot. **B.** Validation of *CREBBP* knockdown by qRT-PCR. **C.** *CREBBP* knockdwon inhibits proliferation in HEY cells. **D.** *CREBBP* knockdown inhibits proliferation in SKOV3 cells. **E.** *CREBBP* knockdown inhibits colony formation. **F.** Statistical analysis of colony numbers.

To investigate the role of CBP in ovarian cancer, two common ovarian cancer cell lines (HEY and SKOV3) were selected and infected with lentivirus harboring CBP shRNA (shCBP-1 and shCBP-2, respectively), and the control cells were infected with shRNA targeting firefly luciferase (shLuc). To detect the knockdown efficiency of shRNA, we collected whole cell lysates for western blot and total RNA for qRT-PCR. As the results shown, the two shRNAs significantly reduced CBP protein level (Fig. 2A) and decreased the level of *CREBBP* mRNA (Fig. 2B). Since CBP is a major transcription co-activator that regulates tumorigenesis, we examined the effect of CBP on proliferation in ovarian cancer cells using CCK-8 assay. The result indicated that *CREBBP* knockdown suppressed tumor proliferation both in HEY and SKOV3 cells (Fig. 2C and 2D). The colony formation assay was utilized to examine the clonogenic potential of cells, and the results also showed that *CREBBP* knockdown inhibited colony formation both in HEY and SKOV3 cells (Fig. 2E and 2F). Taken together, *CREBBP* knockdown significantly suppressed tumor proliferation in ovarian cancer cells.

### *CREBBP* knockdown inhibits tumorigenesis in xenograft tumor model

**Figure 3:**
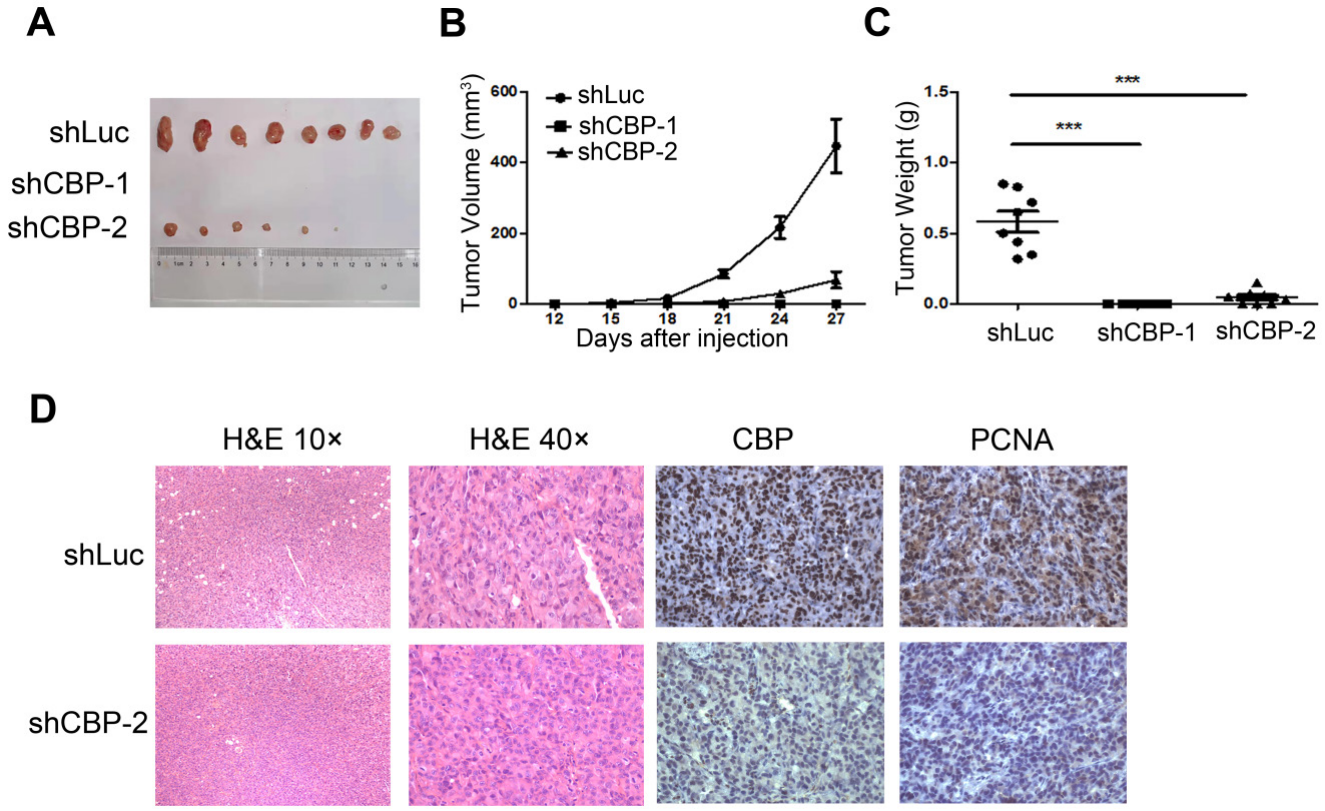
***CREBBP* knockdown inhibits tumorigenesis in xenograft tumor model. A.** Representative images of tumors isolated from the nude mice. **B.** Summary of tumor volumes measured every three days. **C.** Tumor weights in nude mice at the 27^th^ day after inoculation. **D.** Images of hematoxylin and eosin staining and immunohistochemical staining for CBP and PCNA in xenograft tumors.

To investigate the effect of *CREBBP* knockdown on tumorigenesis *in vivo*, a cell line-derived xenograft tumor model was established by subcutaneously injecting *CREBBP* knockdown and the corresponding control cells (HEY shCBP-1, HEY shCBP-2, and HEY shLuc) into the bilateral flanks of BALB/c nude mice. As shown in Figure 3A, knockdown of *CREBBP* by shCBP-1 completely suppressed tumor formation in all mice, and *CREBBP* knockdown by shCBP-2 significantly inhibited tumor growth. Both tumor volumes and tumor weights were dramatically reduced in *CREBBP* knockdown group (Fig. 3B and 3C). Immunohistochemical staining for CBP and proliferation marker PCNA were further performed. As shown in Figure 3D, *CREBB* knockdown cells showed a reduced level of PCNA. Taken together, these results indicated that knockdown of *CREBBP* suppressed tumor growth *in vivo*.

### *CREBBP* knockdown promotes chemo-sensitivity and induces apoptosis in ovarian cancer cells

**Figure 4:**
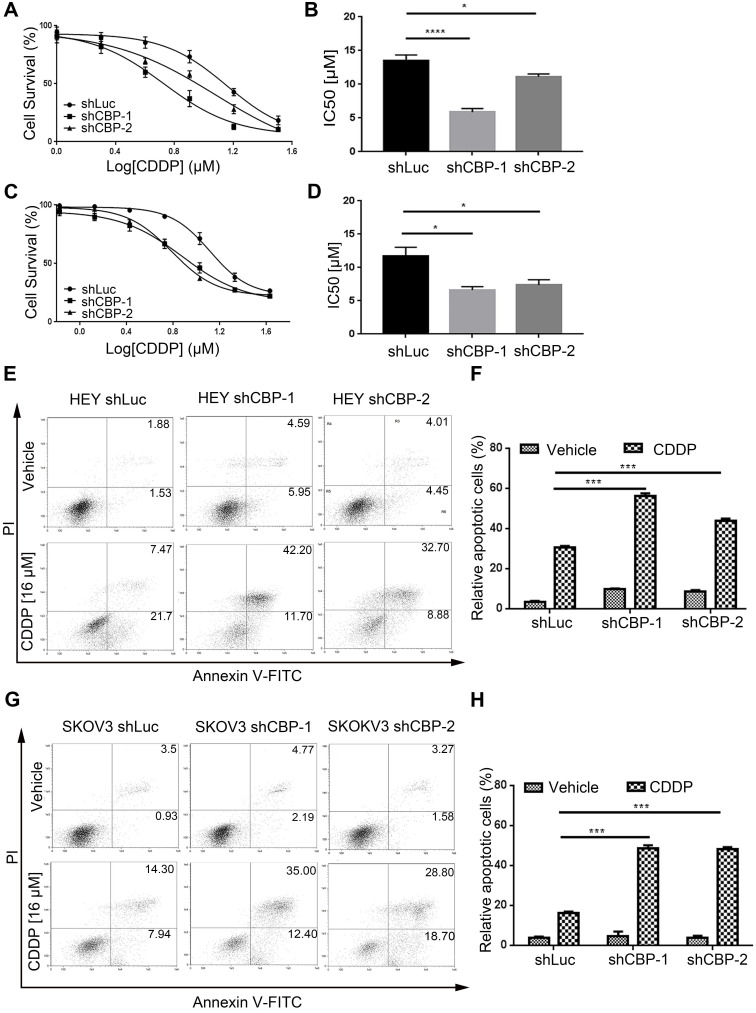
***CREBBP* knockdown promotes chemo-sensitivity and induces apoptosis in ovarian cancer cells. A.** *CREBBP* knockdown promotes chemo-sensitivity in HEY cells. **B.** IC50 values of HEY cells. **C.** *CREBBP* knockdown promotes chemo-sensitivity in SKOV3 cells. **D.** IC50 values of SKOV3 cells. **E.** *CREBBP* knockdown enhances CDDP-induced apoptosis in HEY cells. **F.** Statistical analysis of apoptosis in HEY cells. **G.** *CREBBP* knockdown enhances CDDP-induced apoptosis in SKOV3 cells. **H.** Statistical analysis of apoptosis in SKOV3 cells.

To investigate the effect of CBP on chemo-sensitivity in ovarian cancer cells, we detected the viabilities of cells treated with CDDP at different concentrations. As shown in Figure 4A and Figure 4C, *CREBBP* knockdown enhanced the chemo-sensitivity of cells to CDDP both in HEY and SKOV3 cells (Fig. 4A and 4C). IC50 values were dramatically decreased in *CREBBP* knockdown cells (Fig. 4B and 4D). As apoptosis is crucial in the regulation of cellular proliferation and chemo-sensitivity, Annexin V-PI staining was performed to verify the effect of *CREBBP* knockdown on apoptosis in ovarian cancer cells. As the results shown, *CREBBP* knockdown enhanced CDDP-induced apoptosis in both HEY cells (Fig. 4E and 4F) and SKOV3 cells (Fig. 4G and 4H). Taken together, the results indicated that *CREBBP* knockdown promoted chemo-sensitivity, which was correlated with the enhanced apoptosis in ovarian cancer cells.

### *CREBBP* knockdown attenuates UPR

**Figure 5:**
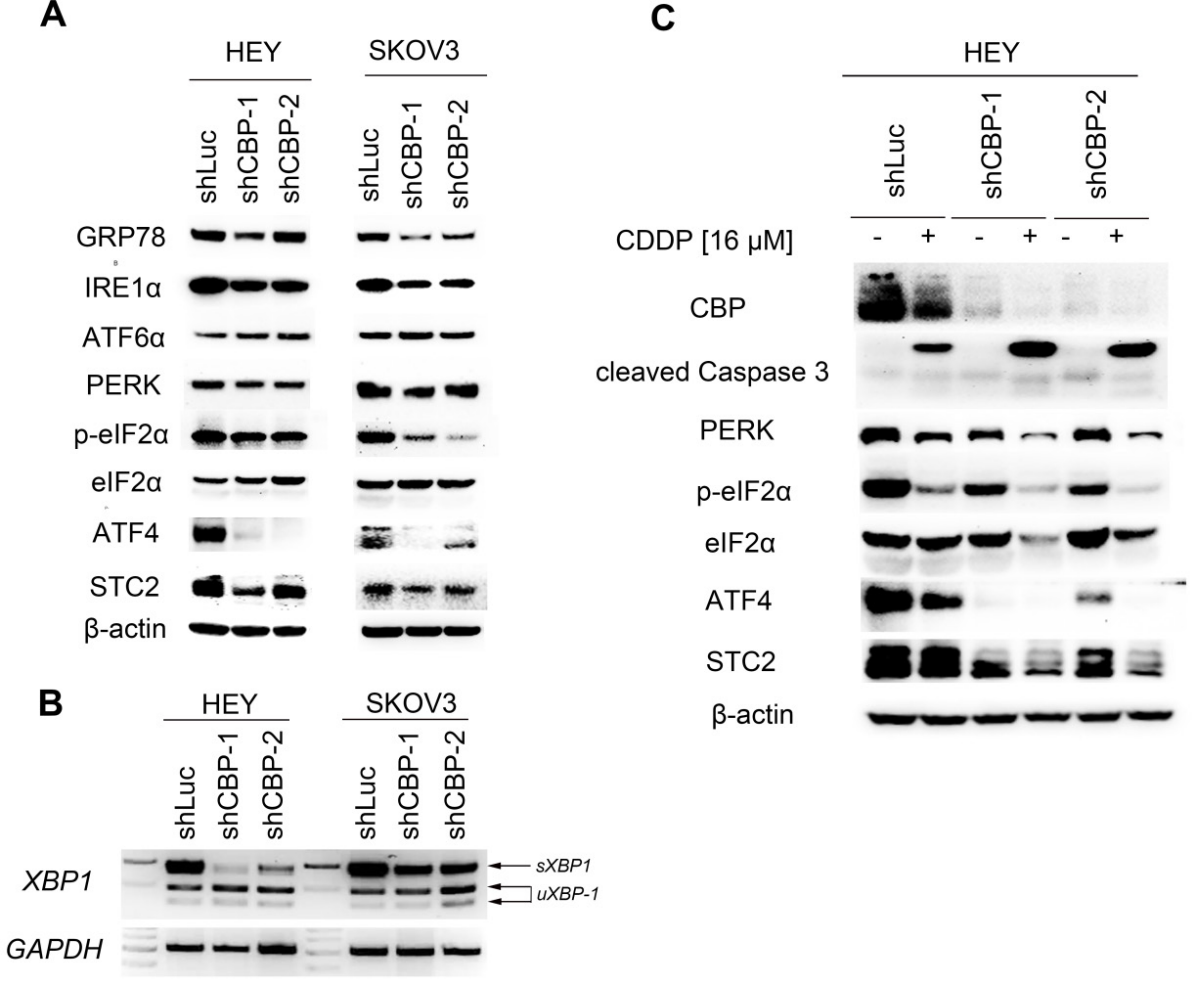
***CREBBP* knockdown attenuates UPR. A.** *CREBBP* knockdown attenuates UPR examined by western blot. **B.** *CREBBP* knockdown reduces the level of *sXBP1* examined by RT-PCR. **C.** PERK signaling pathway is involved in CBP-regulated proliferation and chemo-sensitivity.

To explore the underlying mechanism, we detected the expression of several proteins participating in unfolded protein response (UPR). As shown in Figure 5A, knockdown of *CREBBP* decreased GRP78 expression both in HEY and SKOV3 cells. We further detected proteins involving in the downstream signaling cascade: IRE1α (including IRE1α and XBP1), PERK (including PERK, p-eIF2α, ATF4 and STC2) and ATF6α. *CREBBP* knockdown reduced the expression of IRE1α (Fig. 5A), and the spliced *XBP1* (*sXBP1*) mRNA was also reduced in *CREBBP* knockdown cells (Fig. 5B). Furthermore, expression of PERK, p-eIF2α, ATF4 and STC2 were dramatically decreased in *CREBBP* knockdown cells. However, no obvious change was found in the expression of ATF6α. Our results suggested that IRE1α and PERK signaling pathway were suppressed after *CREBBP* knockdown.

To verify whether UPR signaling pathway was involved in *CREBBP* knockdown-mediated apoptosis, we detected the expression of the aforementioned proteins in CDDP-treated ovarian cancer cells. As shown in Figure 5C, CDDP inhibited CBP expression and induced cleavage of Caspase 3. Moreover, Enhanced Caspase 3 cleavage occurred in CDDP-treated *CREBBP* knockdown cells. The result was in accordance with our previous apoptosis data (Fig. 4E), which indicated that *CREBBP* knockdown indeed promoted CDDP-induced apoptosis. We also found that both PERK, p-eIF2α, ATF4 and STC2 were down-regulated in CDDP-treated cells, and further decreased in *CREBBP* knockdown groups (Fig. 5C). Taken together, these results suggested that *CREBBP* knockdown attenuated UPR, and the PERK signaling pathway was involved in *CREBBP* knockdown-induced apoptosis.

## Discussion

CBP is a crucial transcriptional co-activator in the regulation of gene expression via interacting with other transcription factors, such as SATB2, AP-1 and KLF3 22-24. CBP also acts as a lysine acetyltransferase to regulate protein expressions and functions via direct acetylation e.g., DOT1L, BCAT2, KDM2B and STX17 14-16, 25. Due to the transcription-promoting and LAT activity, CBP is involved in multiple cellular functions and pathological processes especially in tumorigenesis 26-30, but its effect on ovarian cancer has not been fully explored.

Our study showed that high expression of CBP predicted unfavorable prognosis in ovarian cancer patients. Knockdown of *CREBBP* by shRNA significantly suppressed tumor proliferation in ovarian cancer cells both *in vitro* and *in vivo*. Furthermore, *CREBBP* knockdown also enhanced the sensitivity of ovarian cancer cells to CDDP. IRE1α and PERK signaling pathway were suppressed by *CREBBP* knockdown, and the PERK signaling pathway participating in *CREBBP* knockdown-mediated apoptosis.

Emerging evidence showed that activation of UPR signaling contributed to tumor development and was correlated with unfavorable prognosis in cancer patients 31, 32. In EOC, GRP78, ATF6α and PERK were highly expressed in EOC tissues compared to the normal tissues, and correlated with advanced tumor stages. Moreover, high expression of GRP78 predicted poor overall survival (OS), suggesting that expression of UPR-associated proteins could be prognostic biomarkers in ovarian cancer 32. Our study found that knockdown of *CREBBP* decreased GRP78 expression, suggesting low expression of CBP was correlated with good prognosis in ovarian cancer, which was in accordance with the results generated from the Kaplan-Meier Plotter database (Fig. 1).

PERK signaling pathway is the most rapidly activated among the three branches of UPR, followed by ATF6α and IRE1α sequentially 33. STC2 has been reported to be regulated by PERK/ATF4 signaling pathway during UPR and exerts a cytoprotective effect 18. In our study, we demonstrated that knockdown of *CREBBP* dramatically decreased PERK/ATF4/STC2 expression, and promoted apoptosis in ovarian cancer cells treated with CDDP. Previous studies showed that loss of PERK triggered oxidative DNA damage and ROS accumulation thus inhibiting tumorigenesis 34. Therefore, further research is needed to explore whether oxidative stress was involved in CBP loss-induced tumor suppression.

Taken together, our results showed that high expression of CBP was correlated with poor prognosis in patients with ovarian cancer, and knockdown of CBP inhibited tumor growth, promoted chemo-sensitivity, and attenuated UPR in ovarian cancer cells. Our findings indicated that CBP could serve as a novel therapeutic target for ovarian cancer treatment.

## Figures and Tables

**Table 1:** Antibodies lists

Antibodies	Work Dilution	Catalog	Source
CBP	1:1000 for WB; 1:100 for IHC	7389	Cell Signaling Technology, Danvers, MA, USA
GRP78	1:1000 for WB	sc-13968	Santa Cruz Biotechnology, Dallas, TX, USA
ATF6α	1:1000 for WB	sc-166659	Santa Cruz Biotechnology, Dallas, TX, USA
β-actin	1:10000 for WB	MABT825	Sigma-Aldrich, St. Louis, MO, USA
STC2	1:1000 for WB	HPA045372	Sigma-Aldrich, St. Louis, MO, USA
IRE1α	1:1000 for WB	3294	Cell Signaling Technology, Danvers, MA, USA
PERK	1:1000 for WB	5683	Cell Signaling Technology, Danvers, MA, USA
p-eIF2α	1:1000 for WB	3398	Cell Signaling Technology, Danvers, MA, USA
eIF2α	1:1000 for WB	5324	Cell Signaling Technology, Danvers, MA, USA
ATF4	1:1000 for WB	11815	Cell Signaling Technology, Danvers, MA, USA
Cleaved Caspase 3	1:1000 for WB	9661	Cell Signaling Technology, Danvers, MA, USA
PCNA	1:1000 for IHC	ab92552	Abcam, Cambridge, MA, USA
